# Validation of stay-green and stem reserve mobilization QTLs: physiological and gene expression approach

**DOI:** 10.3389/fpls.2025.1541944

**Published:** 2025-02-17

**Authors:** Sukumar Taria, Ajay Arora, Sudhir Kumar, Hari Krishna, Shashi Meena, Biswabiplab Singh, Animireddy China Malakondaiah, Kousalya S., Jasdeep Chatrath Padaria, Pradeep Kumar Singh, Badre Alam, Sushil Kumar, Ayyanadar Arunachalam

**Affiliations:** ^1^ Division of Plant Physiology, Indian Council of Agricultural Research (ICAR)-Indian Agricultural Research Institute, New Delhi, India; ^2^ Indian Council of Agricultural Research (ICAR)-Central Agroforestry Research Institute, Jhansi, UP, India; ^3^ Division of Genetics, Indian Council of Agricultural Research (ICAR)-Indian Agricultural Research Institute, New Delhi, India; ^4^ Indian Council of Agricultural Research (ICAR)-National Institute for Plant Biotechnology, New Delhi, India

**Keywords:** combined stress, QTLs, stay-green, stem reserve mobilization, wheat, yield stability

## Abstract

**Introduction:**

Abiotic stress significantly reduces the wheat yield by hindering several physiological processes in plant. Stay-green (SG) and stem reserve mobilization (SRM) are the two key physiological traits, which can contribute significantly to grain filling during stress period. Validation of genomic regions linked to SG and SRM is needed for its subsequent use in marker-assisted selection in breeding program.

**Methods:**

Using a physiological and gene expression approach, quantitative trait loci (QTLs) for stay-green (SG) and stem reserve mobilization (SRM) were validated in a pot experiment study using contrasting recombinant inbred lines including its parental lines (HD3086/HI1500) in wheat. The experiment was laid down in a completely randomized design under normal (control, drought) and late sown (heat and combined stress) conditions during the 2022-2023 rabi season. Drought stress was imposed by withholding irrigation at the anthesis stage, whereas heat stress was imposed by 1-month late sowing compared to the normal sowing condition. Combined stress was imposed by 1-month late sowing along with restricted irrigation at the flowering stage. Superior lines (HDHI113 and HDHI87) had both SG and SRM traits, whereas inferior lines (HDHI185 and HDHI80) had contrasting traits, i.e., lower SG and SRM traits. HD3086 and HI1500 had SG and SRM traits respectively. Potential candidate genes were identified based on the flanking markers of the mapped QTLs using the BioMart tool in the Ensembl Plants database to validate the identified QTLs. Real-time gene expression was conducted with SG-linked genes in the flag leaf and SRM-linked genes in the peduncle.

**Results and Discussion:**

In this study, HDHI113 and HDHI87 showed higher expression of SG-related genes in the flag leaf under stress conditions. Furthermore, HDHI113 and HDHI87 maintained higher chlorophyll a content of 7.08 and 6.62 mg/gDW, respectively, and higher net photosynthetic rates (P_N_) of 17.18 and 16.48 µmol CO_2_/m^2^/s, respectively, under the combined stress condition. However, these lines showed higher expression of SRM-linked genes in the peduncle under drought stress, indicating that drought stress aggravates SRM in wheat. HDHI113 and HDHI87 recorded higher 1,000-grain weights and spike weight differences under combined stress, further validating the identified QTLs being linked to SG and SRM traits. Henceforth, the identified QTLs can be transferred to developed wheat varieties through efficient breeding strategies for yield improvement in harsh climate conditions.

## 1 Introduction

Wheat is an important staple food crop of humankind; however, its production potential is hindered due to drought, heat, and combined stress (Kumar et al., 2021; Meena et al., 2023). Due to a decline in precipitation and an increase in air temperature, the effects of global warming have become much more noticeable. In addition, crop yield is significantly affected by the frequent combined action of heat and drought stress (Sato et al., 2024). To ensure global food and nutritional security by 2050, there must be a major improvement in the rate of genetic gain in crop yield, quality, input use efficiency, and adaptation to biotic and abiotic challenges (Kumar et al., 2016). Delving into this, it is imperative to develop wheat varieties with increased resilience to abiotic stress. Stay-green (SG) and stem reserve mobilization (SRM) are two important physiological traits that can be targeted for yield improvement in wheat in the current era of global climate change. Thus, the identification of genomic regions linked to SG and SRM and the mining of candidate genes within the quantitative trait locus (QTL) region are of utmost importance for future breeding approaches for yield enhancement in wheat.

SG is a trait in which leaves retain green tissue from flowering to physiological maturity instead of senescing and it is considered an important trait for grain filling (Zhang et al., 2019). SG traits improve the grain yield by improving photosynthetic capacity, retaining higher chlorophyll content, and extending the grain filling period (Reynolds et al., 2000; Kumari et al., 2013; Pinto et al., 2016). It is well known that rubisco is the most abundant protein in the earth. Although both large (*rbcL*) and small subunits of proteins (*rbcS*) are required for the functionality of a protein, the amount of rubisco in plant has been thought to be influenced by the transcript levels of *rbcS* (Kumar et al., 2022). In addition, chlorophyll degradation is a marker of leaf senescence and is catalyzed by six chloroplast-localized chlorophyll catabolic enzymes namely, non-yellow coloring 1(NYC1)/NYC1-like (NOL), 7-hydroxymethyl chlorophyll a reductase (7-HCAR), magnesium dechelatase, pheophytin pheophorbide reductase (PPH), pheophorbide a oxygenase (PAO), and red chlorophyll catabolite reductase (RCCR) (Hörtensteiner and Kräutler, 2011). Furthermore, leaf senescence is also regulated by proteases such as aspartic protease (Kato et al., 2004), leaf nutritional status such as potassium (K^+^) in the flag leaf (Hosseini et al., 2016), and glutamate decarboxylase, a rate-limiting enzyme of GABA (gamma-aminobutyric acid) cycle (Khan et al., 2021). SG is also controlled by the photosystem-II activity of mesophyll cells and antioxidant status (Luo et al., 2013; De Simone et al., 2014). It was demonstrated that Δ-1-pyrroline-5-carboxylate synthase 2 (P5CS2), a proline biosynthetic enzyme, was highly expressed in SG sorghum line than in senescent lines (Johnson et al., 2015). Plant hormones such as cytokinin delay leaf senescence in plants (Wu et al., 2021; Glanz-Idan et al., 2022). Although isopentenyl transferase is the rate-limiting step in cytokinin biosynthesis, lonely guy (LOG) also catalyzes the direct activation of cytokinin biosynthesis (Kuroha et al., 2009), thereby contributing to SG traits.

During stress, stem reserves, particularly fructans, serve as a potential buffer for grain filling, when current leaf photosynthesis is inhibited by various stress (Blum, 1998). Stem reserves can contribute 20% to 40% of the final grain weight in non-stressed environments (Vignjevic et al., 2015), which can rise up to 70% under stressed conditions during grain filling (Rebetzke et al., 2008). The plant hormone ABA enhances the fructans metabolism in wheat (Valluru, 2015; Zhang et al., 2020) and a correlation between the expression of NCED1 and ABA content and the remobilization of stem water-soluble carbohydrates (WSCs) has been well demonstrated (Xu et al., 2016). Furthermore, sucrose non-fermenting-1 related protein kinase1 (SnRK1) regulates the transcriptional network through the phosphorylation of transcription factors in response to sugar starvation and energy stress (Baena-González et al., 2007; Mair et al., 2015). In addition, pentatricopeptide-repeat (PPR) containing protein genes were found to be associated with stem WSCs in bread wheat (Dong et al., 2016), indicating the possible involvement of PPR genes in SRM. The involvement of endoglucanase in stem WSC accumulation and remobilization in wheat was reported by Guerra et al. (2021) using the genome-wide association study (GWAS) approach. However, SG and SRM are two mutually exclusive traits (Blum, 1998) that can be combined to improve the grain filling in wheat under stress conditions. In our previous study, we mapped 11, 2, and 1 QTLs for soil plant analysis development (SPAD) value, leaf senescence rate, and SRM efficiency, respectively, in wheat under stress conditions (Taria et al., 2023).

To our knowledge, functional molecular markers for SG and SRM in wheat are not available. Furthermore, there is a lack of research on breaking the negative linkage between SG and SRM to pyramid these traits to enhance resource availability for grain filling under stress conditions. Moreover, the QTLs for SRM are unmapped for use in marker-assisted selection (MAS). Thus, it is necessary to unveil the genomic regions linked to these traits for further use in MAS. Given the fact mentioned above, four selected lines from our previous study (Taria et al., 2023) including two parental lines (HD3086/HI1500) were used to validate the identified QTLs by employing a physiological approach and *in-vivo* gene expression of QTL-linked genes. Leaf gas exchange studies were conducted using a portable infrared gas analyzer (IRGA) and photosynthetic pigment studies were accomplished using a spectrophotometer. Principal component analysis and clustering analysis were conducted to find out the latent variables and to check the variability in these lines. In this study, it was confirmed that the identified QTLs governed the SG and SRM traits by modulating the expression of the putative candidate genes. The identified genes can be further manipulated by gene editing technologies for further improvement in these traits. Furthermore, the superior lines, HDHI113 and HDHI87, can be used as donor parents for SG and SRM traits in elite wheat cultivars.

## 2 Materials and methods

### 2.1 Plant materials

A total of six lines (HDHI113, HDHI87, HDHI185, HDHI80, HD3086, and HI1500) were grown at a pot culture experimental site at the Division of Plant Physiology, IARI, New Delhi. Four lines (HDHI113, HDHI87, HDHI185, and HDHI80) were selected from our earlier experiment during the 2021-2022 rabi season (Taria et al., 2023). The set of contrasting lines was selected using the multi-trait genotype-ideotype index (MGIDI) across multi-environment stress conditions (Olivoto and Nardino, 2021). HDHI113 and HDHI87 have combined SG and high SRM potential, whereas HDHI185 and HDHI80 have non-SG and lower SRM potential traits. The selected lines were evaluated under normal and late-sown conditions during the 2022-2023 rabi season. Drought stress was imposed at the anthesis stage by withholding irrigation after the booting stage. Heat stress was imposed by 1-month delayed sowing compared to the normal sown condition. In contrast, combined heat and drought (HD) stress was imposed by delayed 1-month sowing with restricted irrigation after the booting stage to impose drought stress at the anthesis stage. The temperature (minimum and maximum temperatures in °C) and rainfall (mm) during the wheat cropping season (2022-2023) are depicted in **Supplementary Figure S1**. The maximum and minimum temperatures at the time of anthesis under the control, drought, heat, and combined stress conditions are given in **Supplementary Table S1**. The average temperature at anthesis in the normal sown condition was 15.5°C, whereas an average temperature of 24.3°C was recorded in the late-sown condition.

### 2.2 Measurement of soil moisture content

The soil moisture content (SMC %) was estimated periodically from the anthesis stage up to physiological maturity under the control, drought, heat, and combined stress conditions. Fresh soil samples of 25g were taken from the rooting zone of potted plants and kept in a hot oven at 105°C. The SMC was calculated according to Faulkner et al. (1989) and calculated as


$$\text{SMC}=\frac{\text{FM}-\text{DM}}{\text{DM}}\;\times 100$$

where FM represents soil fresh mass (g) and DM represents soil dry mass (g).

The SMC at different developmental stages under the control, drought, heat, and combined stress is depicted in **Supplementary Figure S2**.

### 2.3 E-mapping of candidate genes in the identified SG and SRM QTLs

The potential candidate genes were selected from identified QTLs for SG (SPAD and LSR) and SRM traits (Taria et al., 2023). To identify potential candidate genes, the flanking marker was used to search in the Chinese Spring (CS) wheat genome using the BioMart tool, available in the Ensembl Plants database (https://plants.ensembl.org/biomart/martview). The list of selected candidate genes and respective primers are given in **Supplementary Table S2**.

### 2.4 Primer design and quantitative real-time PCR analysis

Primers were designed using the PrimerQuest™ tools available at Integrated DNA Technology (IDT) software (https://www.idtdna.com/PrimerQuest/Home/Index). The primers were designed by selecting quantitative PCR (qPCR) with two primer (intercalating dyes) icons available in IDT software. The selection of the primers was done by checking the hairpin (secondary) structures of the primer list and cross-checking their melting temperature and ΔG value (**Supplementary Table S2**). The amplicon size of the genes was also confirmed using the sequence manipulation suite (SMS) tool (https://www.bioinformatics.org/sms).

Plant flag leaf and peduncle RNA were isolated using a Sigma™ Plant Total RNA kit. For the removal of the trace amount of genomic DNA from the RNA sample, column DNase I digestion was used. The quality and quantification of isolated RNA were checked using 1.2% agarose gel electrophoresis (for integrity) and a spectrophotometer (Nano Drop™ 1000, Thermo Fisher Scientific) respectively. The gel was run for 5V/cm for 60 min.

A Thermo Scientific Verso cDNA synthesis kit was used for the synthesis of complementary DNA (cDNA) from the RNA sample. Anchored Oligo dT primers were used to provide flexible RNA priming for cDNA synthesis. In total, 20µl of the reaction mixture (5X cDNA synthesis buffer, dNTP mix, RNA primer, Verso Enzymes Mix, RNA template, and nuclease-free water) was provided with a thermal cycle of 30 min at 42°C for 1 cycle followed by inactivation for 2 min at 95°C for 1 cycle. To check the amplification of cDNA, a normal PCR reaction was set up with housekeeping gene primers (actin genes).

The real-time quantitative PCR reaction was performed using a Step One Plus TM real-time detection system to determine gene expression under the control, drought, heat, and combined stress conditions and 10µl of reaction mixture (SYBR, Forward primer, Reverse primer, cDNA, High ROX and Nuclease free water) was used in the reaction. The program was set at 95°C for denaturation followed by an annealing temperature of 58°C for primer binding. Finally, 72°C was set for the final extension. Two melt peaks were recorded for two genes put in one plate (24 samples for each gene) for confirmation of the specific gene amplification. Fold changes in gene expression (as 2ΔCt) were calculated by the comparative Ct method (Schmittgen and Livak, 2008), where ΔCt = [Ct target gene - Ct reference gene]. Actin was set as the reference gene (housekeeping gene) for normalization of the gene expression data.

### 2.5 Estimation of leaf chlorophyll and carotenoid concentration

At the anthesis stage, the concentration of chlorophyll and carotenoids in the flag leaves was determined according to the method outlined by Hiscox and Israelstam (1979). 6 mg of fresh leaf sample was added to the test tube containing 6 ml of dimethyl sulfoxide (DMSO). Tubes were kept in the dark for 4 h at 65 °C. The sample was then removed and cooled at room temperature. The absorbance of a known volume of leachates was measured at 663 and 645 nm for the leaf chlorophyll concentration and 480, 649, and 665 nm for the total leaf carotenoid concentration. Chlorophyll a (Chl a), chlorophyll b (Chl b), and total chlorophyll content (Total Chl) were estimated using the formula given by Arnon (1949), while total carotenoid content (Total Car) was determined by following the formula provided by Wellburn (1994) as follows:


$$\text{Chlorophyll a}=\frac{\left[\left(12.7\times {\text{A}}_{663}\right)-\left(2.69\times {\text{A}}_{645}\right)\right]\times \text{V}}{\left(1000\times \text{W}\right)}$$

$$\text{Chlorophyll b}=\frac{\left[\left(22.9\times {\text{A}}_{645}\right)-\left(4.68\times {\text{A}}_{663}\right)\right]\times \text{V}}{\left(1000\times \text{W}\right)}$$

$$\text{Total chlorophyll }=\frac{\left[\left(20.2\times {\text{A}}_{645}\right)+\left(8.02\times {\text{A}}_{663}\right)\right]\times \text{V}}{\left(1000\times \text{W}\right)}$$

For the calculation of the total carotenoids, the following formulae were used


$${\text{C}}_{\text{a}}=\left(12.19\times {\text{A}}_{665}-3.45\times {\text{A}}_{649}\right)$$

$${\text{C}}_{\text{b}}=\left(21.99\times {\text{A}}_{649}-5.32\times {\text{A}}_{665}\right)$$

$$\text{Total carotenoids}=\frac{\left[\left(1000\times {\text{A}}_{480}\right)-\left(2.14\times {\text{C}}_{\text{a}}\right)-\left(70.16\times {\text{C}}_{\text{b}}\right)\right]\times \text{V}}{220\times \text{W}}$$

Whereas,


A480=Absorbance values at 480 nm

A645=Absorbance values at 645 nm

A649=Absorbance values at 649 nm

A663=Absorbance values at 663 nm

A665=Absorbance values at 665 nm

W=Weight of the sample in g

V=Volume of the solvent used (ml)

For dry weight conversion, 1 g of fresh leaf tissue was kept in a hot oven (70 °C) to obtain the dry weight of the respective samples and the chlorophyll and carotenoid concentrations were expressed on a dry weight basis (Dwivedi et al., 2018). The percentage decrease of photosynthetic pigments under stress was calculated with reference to the control condition.

### 2.6 Measurement of gas exchange traits

Gas exchange traits were measured in fully expanded (sunlit exposed) flag leaves using a portable IRGA, model *LI6400XT* (Li-COR Ltd., Lincoln, Nebraska, USA), at the anthesis stage. All the parameters were measured between 10:00 am and 11:00 am by providing an artificial light source of 1,000 µmol (photon) m^-2^ s^-1^ (Dwivedi et al., 2018). The recorded parameters were net photosynthetic rate (P_N_), stomatal conductance to water vapor (g_sw_), transpiration rate (E), and instantaneous water use efficiency (WUE_i_). WUE_i_ was calculated by dividing the net photosynthetic rate (P_N_) by the transpiration rate (E) (Yan et al., 2015; Redhu et al., 2024). The percentage decrease in P_N_ under stress was calculated in reference to the control condition.

### 2.7 1000-grain weight and spike weight difference

For SG traits, we recorded the 1000-grain weight (TGW) of each line at physiological maturity. For SRM traits, all the leaves of the five main culms were defoliated at 12 days after anthesis and the stem weight was recorded. The remaining five defoliated culms were left in the field and were sampled at physiological maturity by allowing the mobilization of stem reserve from stems to grains. Spike weight difference (SWD) was calculated by adopting the methods described by Ehdaie et al. (2006) as follows:


$$\text{Spike weight difference }=\frac{\text{Spike weight at physiological maturity}-\text{Spike weight at }12\text{ days after anthesis}}{\text{Spike weight at physiological maturity}}\times 100$$

### 2.8 Statistical analysis

The least significant difference (LSD) test was conducted to check significant differences among the lines at α = 0.05 (n=5). Principal components were extracted using the *FactomineR* package and enhanced visualization was carried out using the *factoextra* package. Agglomerative clustering was performed using Euclidean distance measures. A hierarchical rectangular dendrogram was created using the *hclust* function by following the agglomeration methods of Ward.D2. In addition, the cluster score was calculated as a weighted linear combination of physiological traits (i.e., summation of weightage multiplied by their respective physiological trait), where X_i_ referred to the mean value of the i_th_ physiological trait of a given cluster and W_i_ referred to the weightage associated with the i_th_ physiological trait of a given cluster. The weightage was obtained from the commonalities in the PCA analysis. The cluster score was calculated by the methods described by Nagar et al. (2015). Figures were created using the inbuilt *ggplot2* package in R. Values are presented in the graphs as the means of traits, whereas vertical bars represent standard error.

The gene expression of candidate genes was visualized through a heat map using the “pheatmap” package in R. A spider network chart was prepared using the “fmsb” package in R.

## 3 Results

### 3.1 Gene expression analysis of potential candidate genes

**Figure 1:**
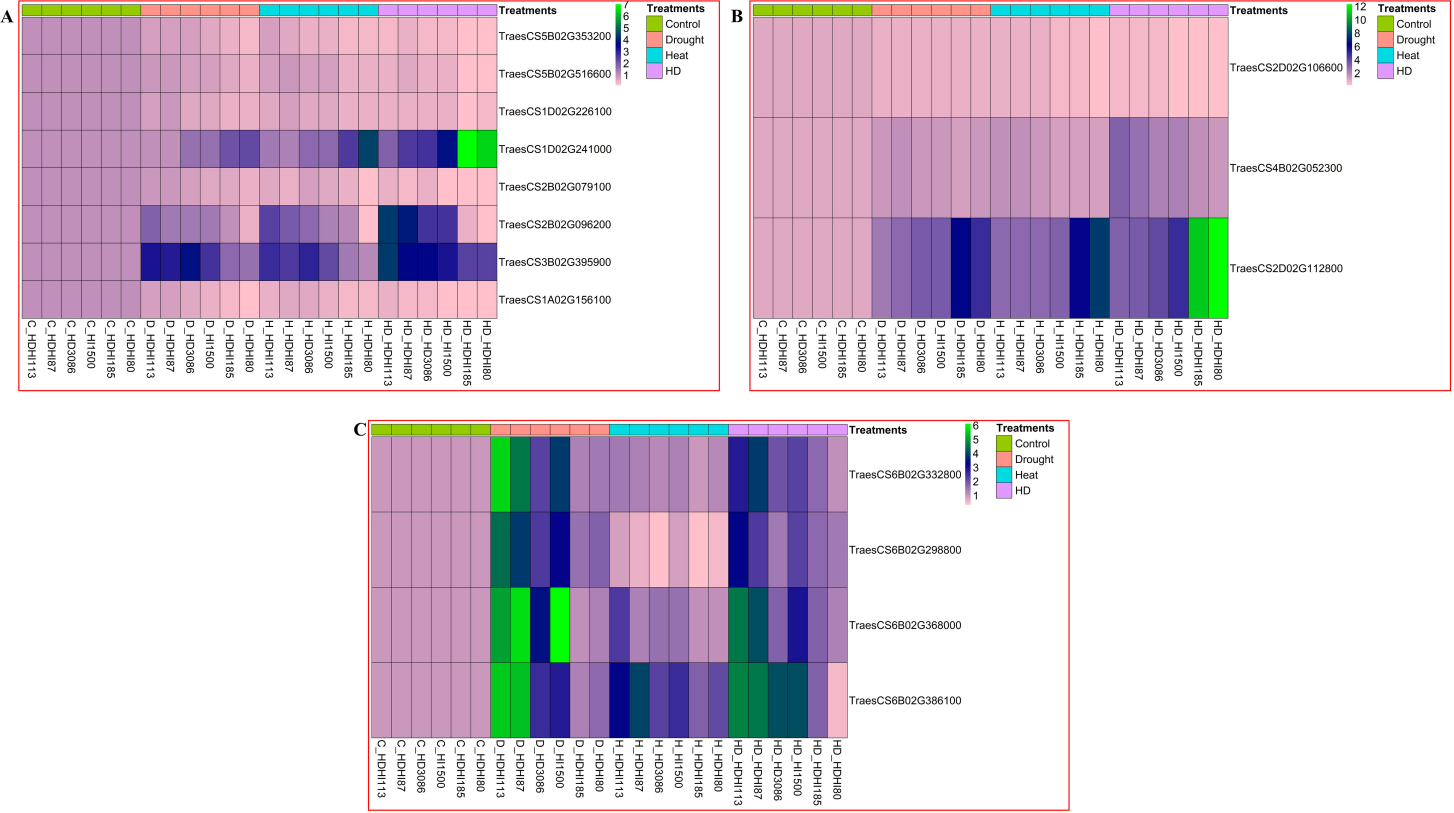
Relative gene expression of stay-green-related genes **(A)**, leaf senescence-related genes **(B)**, and stem reserve mobilization-related genes **(C)** under drought, heat, and combined stress conditions.

The RT-PCR analysis revealed that the superior lines, HDHI113 and HDHI87, showed a higher expression of photosynthesis-related genes such as a LHCII-type chlorophyll a-b binding protein (*TraesCS5B02G353200*), PSB28 protein (*TraesCS5B02G516600*), chlorophyll synthase (*TraesCS1D02G226100*), and rubisco small subunit (*TraesCS2B02G079100*). These lines also recorded a higher expression of L-ascorbate peroxidase (*TraesCS2B02G096200*), pyrroline carboxylate synthase (*TraesCS3B02G395900*), and cytokinin riboside 5’-monophosphate phosphoribohydrolase LOGL10 (*TraesCS1A02G156100*) under the combined stress condition. Furthermore, the superior RILs exhibited a lower expression of the *TraesCS1D02G241000* gene, encoding 7-HCAR, compared to the inferior RILs under the combined stress condition (**Figure 1A**). Expression analysis of senescence-associated genes were also studied. The superior RILs, HDHI113 and HDHI87, showed a lower expression of aspartyl protease family protein 2-like (*TraesCS2D02G112800*) compared to the inferior RILs and parents under combined stress conditions. Furthermore, expression of potassium transporter-9 (*TraesCS2D02G106600*) and glutamate decarboxylase1-like (*TraesCS4B02G052300*) was found to be higher in the superior RILs under the drought, heat, and combined stress conditions (**Figure 1B**).

Gene expression analysis of putative candidate genes for SRM was also conducted in the stem peduncle at 12 DAA under the multi-environment stress condition. In this study, we found a higher expression of PPR5 (*TraesCS6B02G332800*), endoglucanse-8-like (*TraesCS6B02G368000*), serine-threonine protein kinase-OSR1-like (*TraesCS6B02G386100*), and NCED1 (*TraesCS6B02G298800*) in the superior lines (HDHI113 and HDHI87) under the drought stress condition followed by the combined stress, control, and heat stress conditions, respectively (**Figure 1C**).

From the gene expression study, it was observed that the superior lines had a higher expression of SG-related genes and a lower expression of 7-HCAR under the drought, heat, and combined stress conditions. However, the superior lines had higher transcript levels of SRM-related candidate genes under the drought stress condition, indicating that drought stress aggravated the mobilization of stored carbon to grains.

### 3.2 Effect of drought, heat, and combined stress on photosynthetic pigments

**Figure 2:**
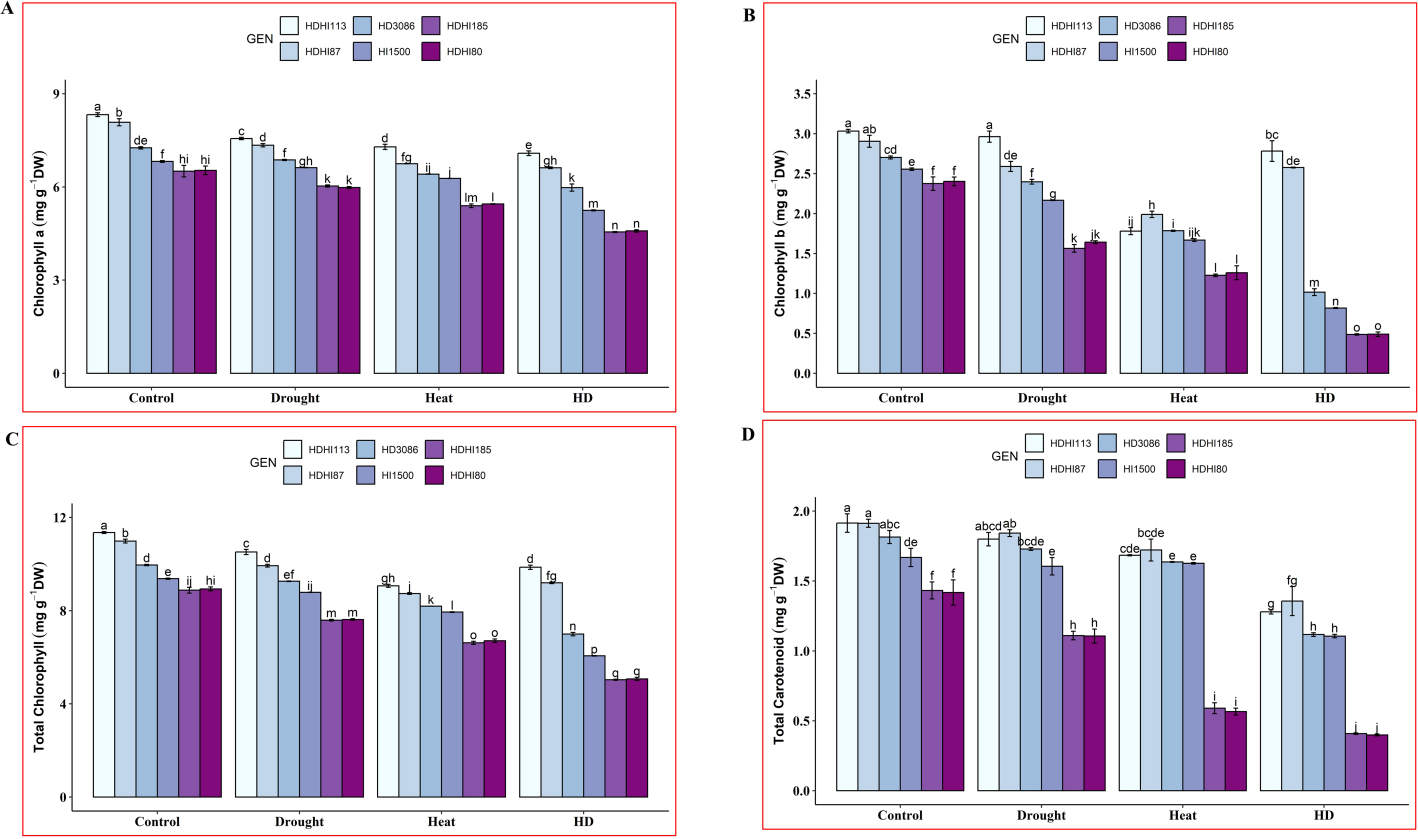
Effect of drought, heat, and combined stress on chlorophyll a **(A)**, chlorophyll b **(B)**, total chlorophyll **(C)**, and total carotenoid **(D)** at the anthesis stage. Different letters indicate significant genotype × treatment interactions among the lines using the LSD test at P<0.05.

Lower concentrations of Chla, Chlb, total Chl, and total Car were observed under drought, heat, and combined stress, respectively, compared to the control condition at the anthesis stage in wheat for all the lines tested, including the parental lines (**Supplementary Table S3**). However, a lower level of leaf photosynthetic pigment was observed under combined stress compared to individual stress. A significant difference in photosynthetic pigment was observed between the superior and inferior lines across all the environmental conditions. Under combined stress, HDHI113 recorded the highest chlorophyll a concentration of 7.08 mg/gDW (14.91% decrease), whereas HDHI185 recorded the lowest chlorophyll a concentration of 4.55 mg/gDW (30.09% decrease) (**Figure 2A**). For chlorophyll b, HDHI113 recorded the highest chlorophyll b concentration of 2.78 mg/gDW (8.24% decrease), whereas HDHI185 and HDHI80 recorded the lowest chlorophyll b concentration of 0.49 mg/gDW (79.53% and 79.58% decreases respectively) under the combined stress condition (**Figure 2B**). For total chlorophyll, HDHI113 recorded the highest total chlorophyll concentration of 9.86 mg/gDW (13.14% decrease), whereas HDHI185 recorded the lowest total chlorophyll concentration of 5.03 mg/gDW (43.31% decrease) under the combined stress condition (**Figure 2C**). For total carotenoid, HDHI113 recorded the highest total carotenoid concentration of 1.36 mg/gDW (29.14% decrease), whereas HDHI80 recorded the lowest total carotenoid concentration of 0.40 mg/gDW (71.86% decrease) under combined heat and drought stress (**Figure 2D**). Furthermore, one of the parents, HD3086, recorded higher photosynthetic pigments at the anthesis stage across multi-environment stress conditions, indicating potential SG traits.

From the photosynthetic pigment study, it was found that HDHI113 and HDHI87 maintained higher chlorophyll and carotenoid pigment levels under combined stress conditions.

### 3.3 Effect of drought, heat, and combined stress on gas exchange traits

**Figure 3:**
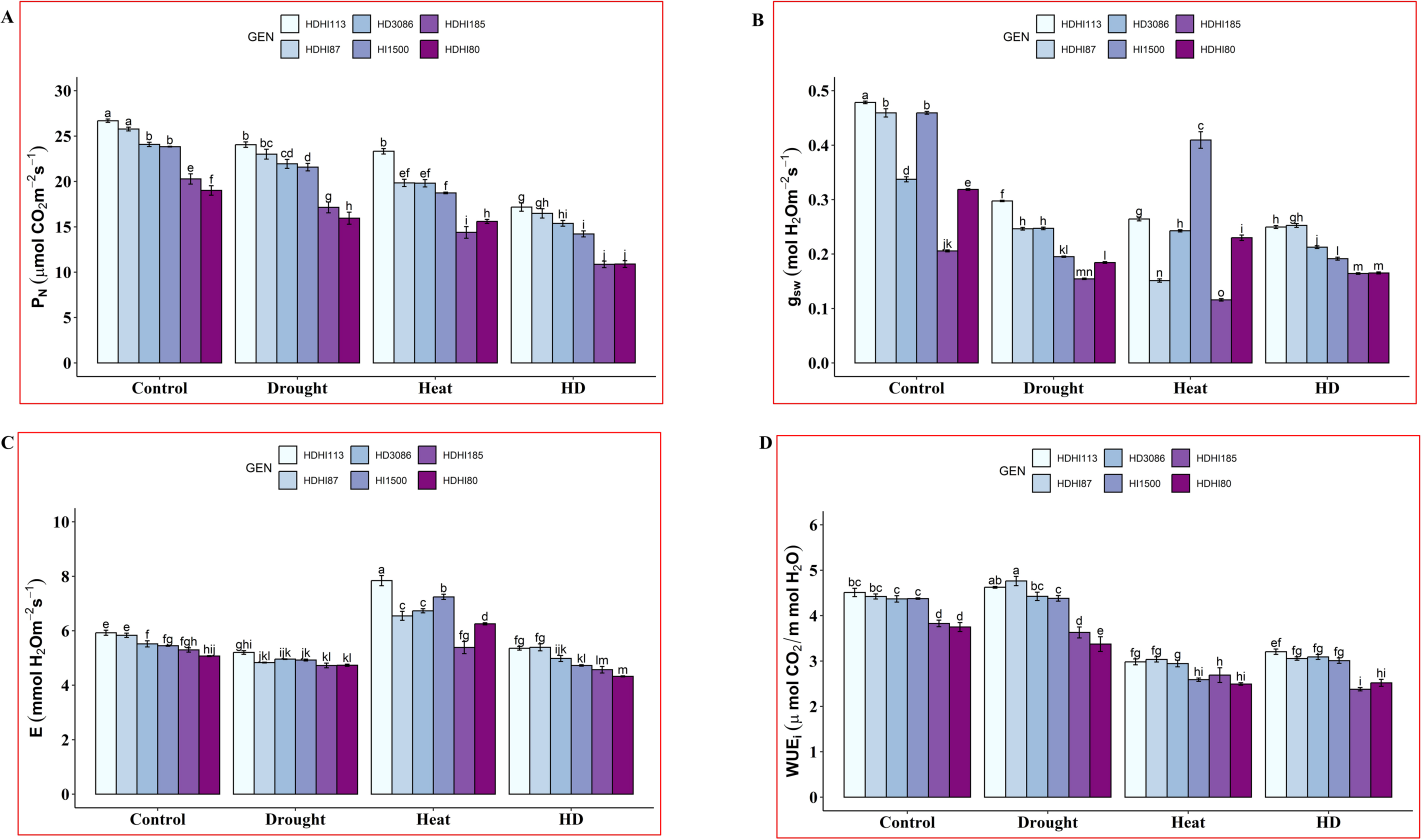
Effect of drought, heat, and combined stress on net photosynthetic rate (P_N_) **(A)**, stomatal conductance (g_sw_) **(B)**, transpiration rate (E) **(C)**, and instantaneous water use efficiency (WUEi) **(D)** at the anthesis stage. Different letters indicate significant genotype × treatment interactions among the lines using the LSD test at P<0.05.

In our study, significant differences in gas exchange traits were noted between the superior and inferior lines across all the environmental conditions (**Supplementary Table S4**). We observed a significant decrease in the P_N_ under combined stress conditions compared to the control, drought, and heat stress conditions (**Figure 3A**, **Supplementary Table S4**). HDHI113 maintained a P_N_ of 17.18 µmol CO_2_ m^-2^ s^-1^ (35.66% decrease) and HDHI185 recorded the lowest P_N_ of 10.87 µmol CO_2_ m^-2^ s^-1^ (46.41% decrease) at the anthesis stage under combined stress conditions. For g_sw_, we observed a significant decrease in g_sw_ in all the lines under stress conditions compared to the control plants (**Figure 3B**, **Supplementary Table S4**). Under combined stress conditions, the superior lines (HDHI113 and HDHI87) maintained a higher g_sw_ (0.25 mol H_2_O m^-2^ s^-1^) and HDHI185 (inferior line) maintained the lowest g_sw_ (0.16 mol H_2_O m^-2^ s^-1^).

E declined under drought stress in all the RILs including the parental lines (**Figure 3C**, **Supplementary Table S4**). Under heat stress conditions, the transpiration rate was enhanced in all the RILs compared to the control condition. The highest E of 7.84 mmol H_2_O m^-2^ s^-1^ was recorded by HDHI113 and the lowest E of 5.38 mmol H_2_O m^-2^ s^-1^ was recorded by HDHI185 under heat stress conditions. Under combined stress conditions, the highest E of 5.39 mmol H_2_O m^-2^ s^-1^ was recorded by HDHI87 and the lowest E of 4.32 mmol H_2_O m^-2^ s^-1^ was recorded by HDHI80. WUE_i_ was enhanced for the superior lines, HDHI113 and HDHI87 under drought stress. However, it declined for the inferior lines, HDHI185 and HDHI80 (**Figure 3D**, **Supplementary Table S4**). Under heat stress conditions, WUE_i_ was found to be decreased in all the tested lines compared to the control and drought stress conditions. However, HDHI87 recorded a higher WUE_i_ (3.04 µmol CO_2_/mmol H_2_O) and HDHI80 recorded the lowest E of 2.49 µmol CO_2_/mmol H_2_O under heat stress conditions. Moreover, a higher WUE_i_ was recorded by HDHI113 (3.21 µmol CO_2_/mmol H_2_O) and the lowest E of 2.38 µmol CO_2_/mmol H_2_O was recorded by HDHI185 under combined stress conditions.

From the gas exchange traits study, it was observed that HDHI113 and HDHI87 maintained higher P_N_ under combined stress conditions.

### 3.4 1000-grain weight and spike weight difference under combined stress

**Figure 4:**
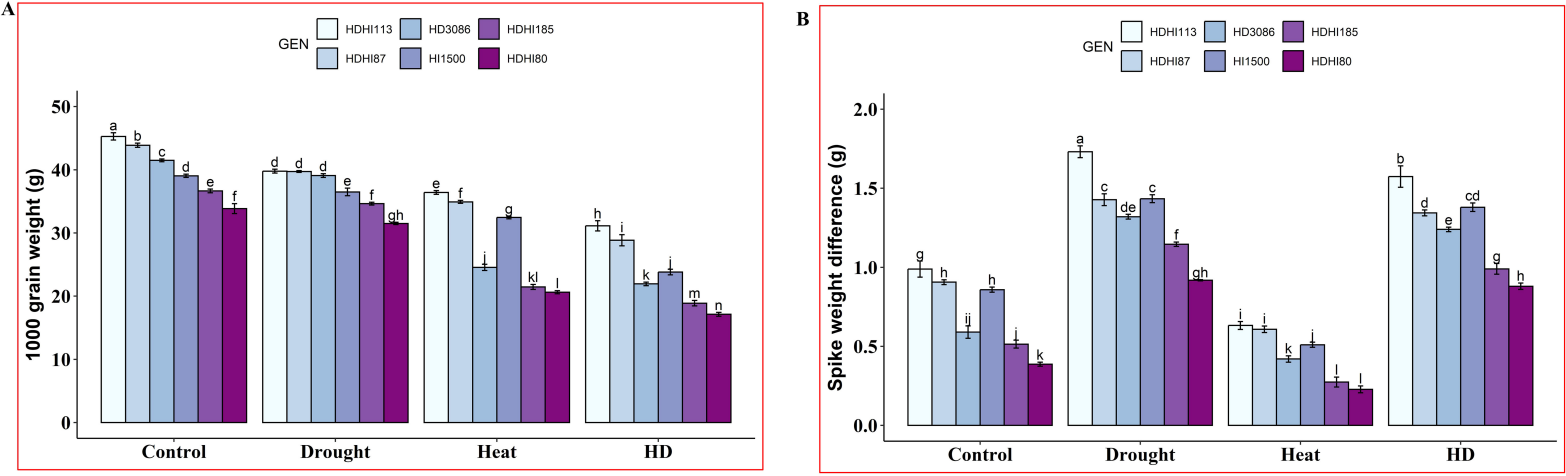
Effect of drought, heat, and combined stress on 1000-grain weight (TGW) **(A)** and spike weight difference (SWD) **(B)** in wheat RILs including the parental lines. Different letters indicate significant genotype × treatment interactions among the lines using the LSD test at P<0.05.

Combined stress significantly reduced the TGW in all the RILs including the parents (**Figure 4A**). Moreover, HDHI113 recorded the highest TGW of 31.13 g and HDHI80 recorded the lowest TGW of 17.12 g under combined stress conditions (**Supplementary Table S4**). Furthermore, drought stress accelerated SRM to grains in all the RILs and HDHI113 recorded the highest SWD of 1.73 g under drought stress (**Figure 4B**, **Supplementary Table S4**) due to the higher SRM. However, heat stress resulted in a lower SWD in all the RILs.

Based on the SG traits, it was found that HDHI113 and HDHI87 recorded higher TGW under all environmental stress conditions. However, these lines recorded higher SWD under drought stress conditions based on SRM traits.

### 3.5 Relative performances of the lines under combined stress conditions

**Figure 5:**
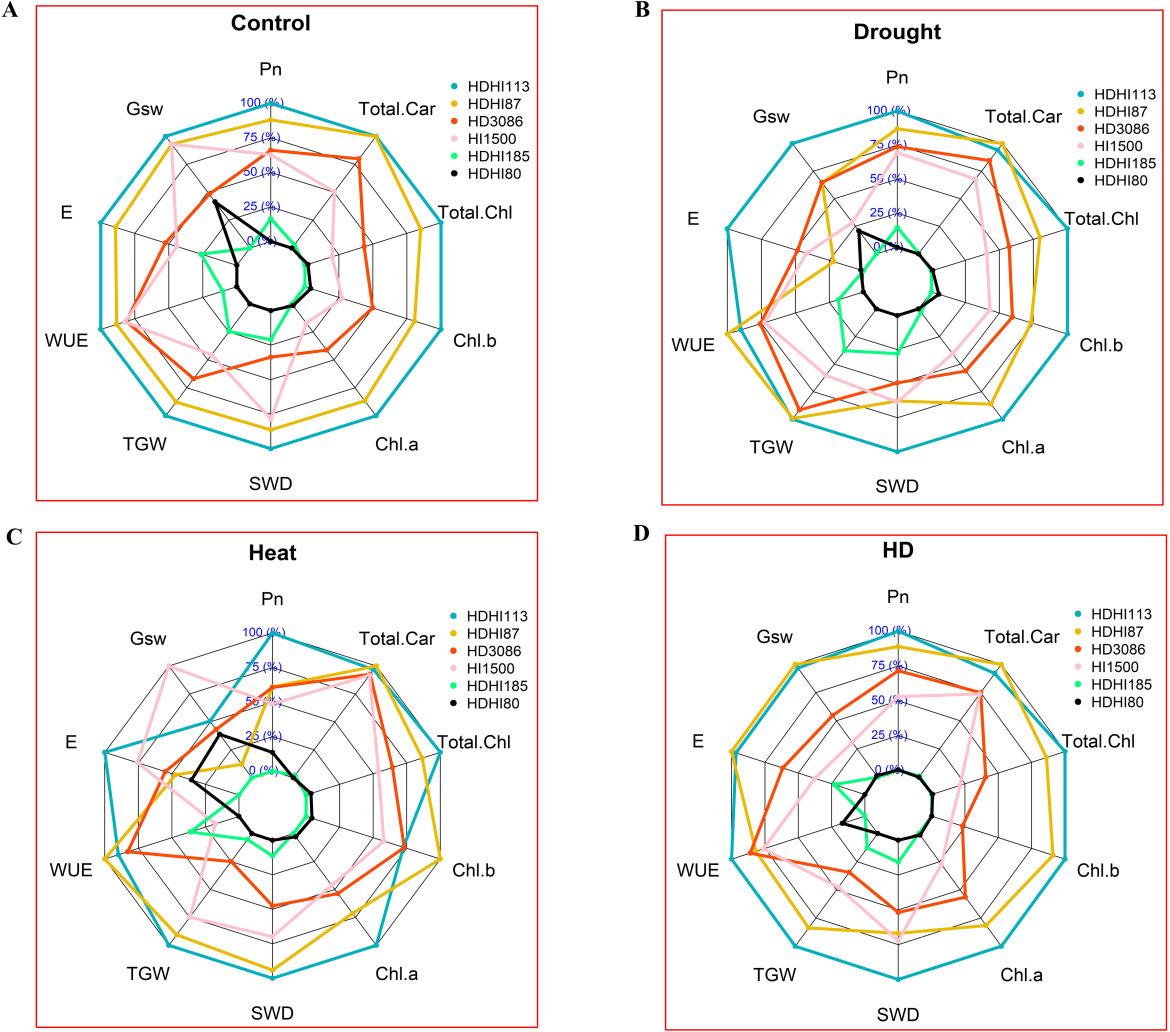
Relative performance of RILs and parents under control **(A)**, drought **(B)**, heat **(C)**, and combined stress conditions **(D)**. Pn, net photosynthetic rate; Gsw, stomatal conductance; **(E)**, transpiration rate; WUE, water use efficiency; TGW, 1000-grain weight; SWD, spike weight difference; Chl a, chlorophyll a; Chl b, chlorophyll b; Total Chl, total chlorophyll; Total Car, total carotenoid.

A comparison of the performance of the RILs including the parents revealed that there were differences in the trait values under the control, drought, heat, and combined stress conditions. With improved gas exchange and photosynthetic pigment characteristics, including grain yield, as shown in the spider network chart, HDHI113 and HDHI87 performed better in multi-environment conditions (**Figures 5A-D**).

### 3.6 Principal component analysis

**Figure 6:**
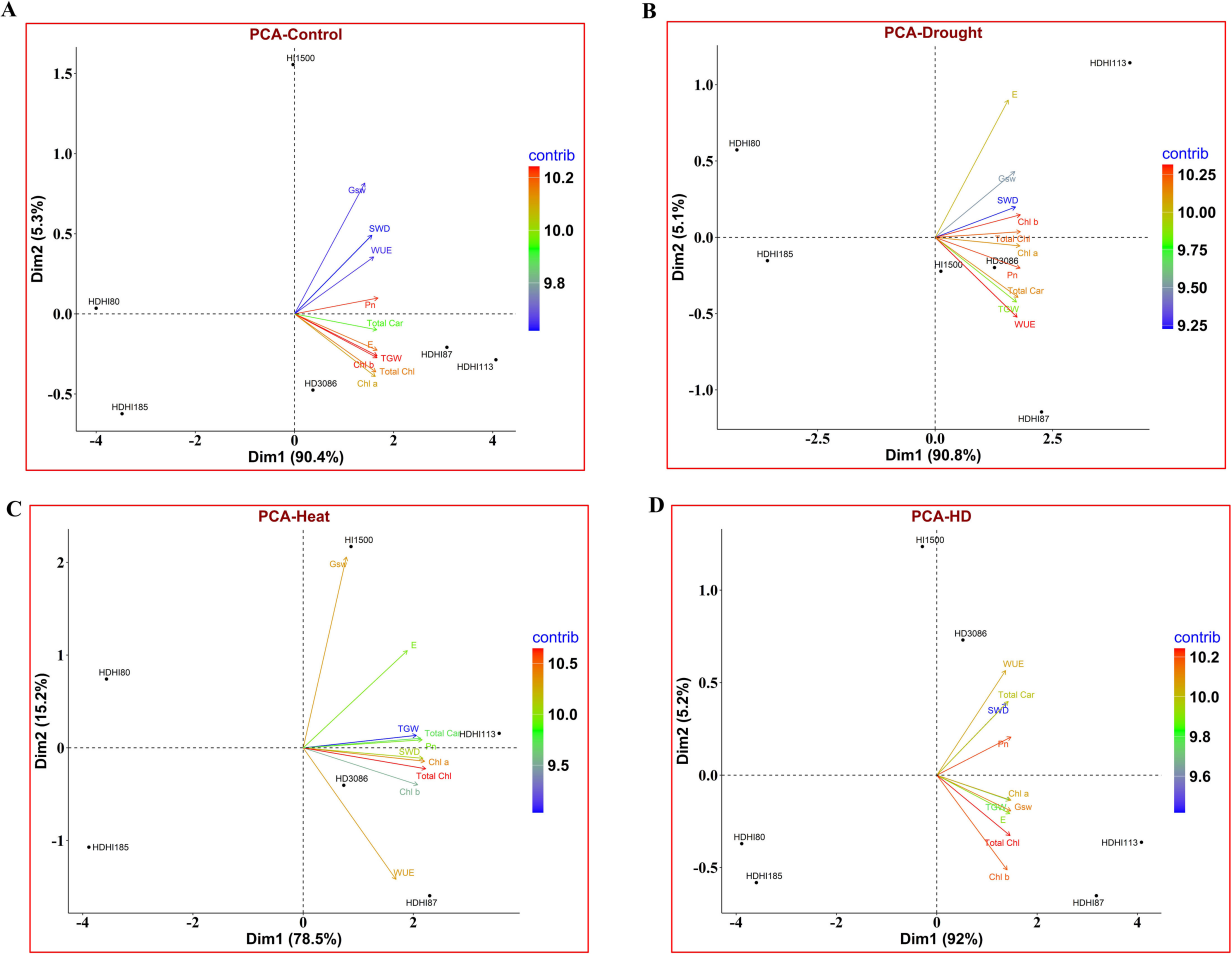
PCA biplot depicting the contribution of traits to Dim1 and Dim2 in the control **(A)**, drought **(B)**, heat **(C)** and combined stress conditions **(D)**. Pn, net photosynthetic rate; Gsw, stomatal conductance; E, transpiration rate; WUE, water use efficiency; TGW, 1000-grain weight; SWD, spike weight difference; Chl a, chlorophyll a; Chl b, chlorophyll b; Total Chl, total chlorophyll; Total Car, total carotenoid.

Using PCA, it was inferred that most of the traits were captured by Dimension 1 (Dim1) under control (**Figure 6A**), drought (**Figure 6B**), heat (**Figure 6C**), and combined stress conditions (**Figure 6D**). Under control conditions, traits including P_N_, TGW, E, Chl b, Total Car, Total Chl, and Chl a were captured by Dim1 (**Supplementary Figure S3A**). Under drought stress conditions, the maximum numbers of traits were captured by Dim1 except for TGW, SWD, Gsw, and E (**Supplementary Figure S3B**). Similarly, all the important traits were captured by Dim1 except for E, Gsw, and WUE under heat stress (**Supplementary Figure S3C**) and total Car, Chlb, SWD, and WUE under combined stress (**Supplementary Figure S3D**).

As most of the traits were captured by Dim1, it can be inferred that these traits significantly contributed towards the grain filling in wheat.

### 3.7 Cluster analysis

**Figure 7:**
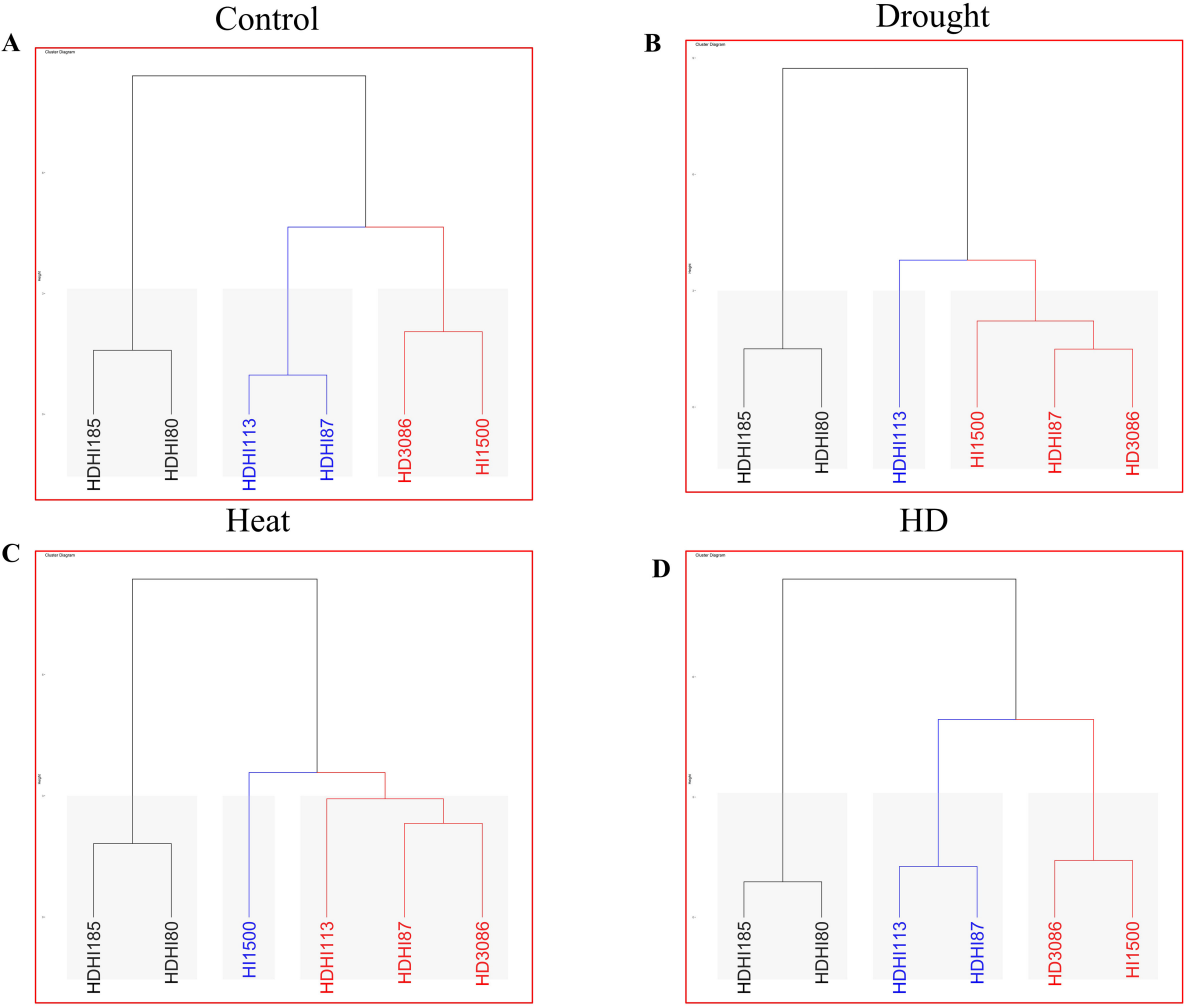
Agglomerative hierarchical clustering depicting various clusters under control **(A)**, drought **(B)**, heat **(C)**, and combined stress **(D)** conditions.

Under each environmental condition, three clusters were formed. Under control conditions, each group contained two lines (**Figure 7A**). Group 1 retained HDHI113 and HDHI87 with the highest cluster score of 106.389 (**Supplementary Table S5A**). Under drought stress conditions, cluster sizes of 1, 3, and 2 were recorded in clusters I, II, and III respectively (**Figure 7B**). Group 1 retained only HDHI113 with the highest cluster score of 95.974 (**Supplementary Table S5B**). Under heat stress conditions, cluster sizes of 3, 1, and 2 in clusters I, II, and III respectively were recorded (**Figure 7C**). Group 1 retained HDHI113, HDHI87, and HD3086 with the highest cluster score of 77.258 (**Supplementary Table S5C**). Under combined stress conditions, each group contained two lines (**Figure 7D**). Group 1 retained HDHI113 and HDHI87 with the highest cluster score of 76.872 (**Supplementary Table S5D**).

## 4 Discussion

One of the main aims of wheat breeding globally is the development of stress-tolerant cultivars; this is particularly important for rain-fed conditions in India. The broadening of genetic diversity by employing diverse alleles for SG and SRM traits is needed for further improvements in wheat yield. Previous studies have reported numerous QTLs for SG traits, however, genomic regions linked to SRM and its potential candidate genes have not been explored so far. In this study, we validated the mapped QTLs by employing a gene expression and physiological approach.

### 4.1 Stay-green traits

In our study, we found a higher expression of SG-related genes such as chlorophyll synthase, chlorophyll a/b binding protein, Psb28, L-ascorbate peroxidase, pyrroline carboxylate synthase, and LOG10 in the superior lines under stress conditions. This indicated the influence of true positive SG-QTLs in maintaining the leaf greenness in the superior lines. It is well-known that chlorophyll a/b binding proteins are part of light-harvesting complexes (LHCs) and genes encoding for LHCs (*Lhca* and *Lhcb*) are of great importance for maintaining photosynthetic activity (Standfuss et al., 2005; Sato et al., 2009). In addition, Psb28 has been shown to protect the RC47 assembly intermediates of PSII and its absence impaired PSII recovery after photodamage in high-temperature and high-light conditions (Weisz et al., 2017). Through transcriptomic analysis, it was also found that the pyrolline carboxylate synthase 2 (P5CS2) gene was highly expressed in SG lines compared to senescent lines in sorghum (Johnson et al., 2015). It has been established that the application of 6-benzylaminopurine improved the grain yield and quality of wheat under water deficit conditions (Zarea and Karimi, 2023). The role of APX in inducing cold tolerance (Xu et al., 2014), heat tolerance (Zhang et al., 2023), and ROS detoxification (Guan et al., 2015; Yan et al., 2016; Shafi et al., 2017) is well reported.

Again, the higher expression of chlorophyll synthase in the superior lines (HDHI113 and HDHI87) was accompanied by higher levels of photosynthetic pigments under stress conditions. However, we found a lower level of photosynthetic pigments under combined stress. Our finding was in line with other studies in wheat (Zaefyzadeh et al., 2009; Sharifi and Mohammadkhani, 2016; Dwivedi et al., 2018; Yousaf et al., 2022), chickpea (Mafakheri et al., 2010), and tomato (Raja et al., 2020). Furthermore, we observed a lower level of photosynthetic pigments in wheat at the anthesis stage under heat stress compared to drought stress (Lamaoui et al., 2018; Yousaf et al., 2022). This greater decrease in leaf chlorophyll under heat stress in the sensitive lines was also probably because of an increase in proteolytic enzyme activity (Al-Khatib and Paulsen, 1984; Harding et al., 1990) and due to the damage to the thylakoid membrane and PSII complex (Ristic et al., 2007). Moreover, retention of chlorophyll content during post-flowering heat stress was reported to minimize yield losses in winter wheat (Fu et al., 2023).

In our study, we also studied the expression patterns of senescence-associated genes. We found a higher expression of 7-HCAR and aspartyl protease in the inferior lines (HDHI185 and HDHI80) under combined stress conditions. This higher expression was also substantiated by lower photosynthetic pigments in these inferior lines. The overexpression of 7-HCAR from cucumber in tobacco has been shown to hasten the dark-induced degradation of chlorophyll (Liu et al., 2021). However, we found a lower expression of K^+^-transporter-9 and glutamate decarboxylase genes in the inferior lines. The role of potassium in inhibiting drought-induced leaf senescence by promoting ABA degradation in barley has been reported (Hosseini et al., 2016). Furthermore, *Arabidopsis thaliana*, which produces less GABA, exhibited a greater vulnerability to drought stress (Mekonnen et al., 2016). Furthermore, it was also reported that the application of GABA to *Oryza sativa* seedlings, creeping bent grass, and *Piper nigrum* improves the performance of the individuals under heat and drought stress conditions respectively (Nayyar et al., 2014; Li et al., 2016; Vijayakumari and Puthur, 2016).

We also found greater downregulation of rubisco small subunit expression under stress conditions in the inferior lines. Rubisco is an important protein for photosynthetic carbon fixation (Ogren and Bowes, 1971; Le Roux et al., 2020). Amongst the senescence downregulated genes (SDGs), the rubisco small subunit (*rbcs*) and chlorophyll a/b binding (*cab*) proteins are mainly downregulated during the senescence process (Rampino et al., 2006). This lower expression of rubisco small subunit in the inferior lines was correlated with a lower P_N_ value under stress conditions. In the present study, a decrease in P_N_ under drought stress was due to stomatal limitation, i.e., we observed a lower g_sw_ in our study (Centritto et al., 2003; Flexas et al., 2004; Grassi and Magnani, 2005; Erismann et al., 2008; Chaves et al., 2009; Peeva and Cornic, 2009), and non-stomatal limitation, i.e., lower chlorophyll content and lower rubisco activity (Xu et al., 2010; Simova-Stoilova et al., 2020). This decrease in g_sw_ was probably due to lower soil moisture content (18.20%) at the anthesis stage (**Supplementary Figure S2**), which induced stomatal closing as a drought avoidance response (Chaves et al., 2009; Ghotbi-Ravandi et al., 2014). The decrease in g_sw_ was substantiated by a decrease in E. The lowering of E under drought stress was reported by various researchers in wheat (Li et al., 2017; Itam et al., 2021; Guizani et al., 2023), naked oat (Zhang et al., 2022), and cowpea (Singh and Reddy, 2011). An increase in WUE_i_ in the superior lines under drought stress was reported by previous researchers in wheat (Van den Boogaard et al., 1997; Sikder et al., 2016) and cowpea (Anyia and Herzog, 2004) as well. However, we observed a greater decrease in P_N_ under combined stress than heat stress at the anthesis stage. In C_3_ plants such as wheat, an increase in temperature above the optimal growth temperature generally decreases P_N_ (Berry and Bjorkman, 1980). In our study, a decrease in P_N_ under heat stress was due to lower g_sw_, except for HI1500 and HDHI80 (as compared to drought stress), lower chlorophyll content, and lower rubisco activity. However, a decrease in g_sw_ under heat stress was decoupled from increased E and lower WUE_i_. Moreover, higher E was observed in HDHI113 under heat stress, probably to make the canopy cool (Ayeneh et al., 2002; Gommers, 2020), and it has been reported that heat-tolerant lines with higher yields exhibited better cooling capacity (Reynolds et al., 1997). Moreover, greater E under heat stress was also because a higher VPD is a natural consequence of higher temperatures (Sharma et al., 2015) and higher stomatal conductance, as transpiration rate can be approximated as VPD×stomatal conductance (Cowan, 1977; Will et al., 2013). Increased E under heat stress was also reported in wheat (Sharma et al., 2015; Nagar et al., 2021). Moreover, decreases in WUE_i_ under heat stress were probably because of higher E compared to the control condition (Sharma et al., 2015) and a decrease in P_N_ (Agarwal et al., 2021), which was the case in our findings. The decrease in P_N_ under combined stress was due to a lowering of g_sw_, lower chlorophyll content, and lower rubisco activity (Kumar et al., 2021; Meena et al., 2023). A decrease in g_sw_ was reported by various researchers in plants under combined stress (El Habti et al., 2020; MaChado and Paulsen, 2001; Shah and Paulsen, 2003). It was reported that the maintenance of E during combined stress is indispensable for sustaining wheat productivity (El Habti et al., 2020). According to Zandalinas et al. (2016), the ability of citrus trees to withstand the combined effects of heat and drought was facilitated by an increase in E. The higher WUE_i_ in the superior lines under combined stress was probably due to the better trade-off mechanisms of the superior RILs leading to the maximizing of carbon assimilation and reducing water loss through transpiration (El Habti et al., 2020).

From this study, it was inferred that HDHI113 and HDHI87 maintained their SG traits under stress conditions by upregulating the SG-linked gene expression to maintain leaf greenness and by activating ROS scavenging systems such as ascorbate peroxidase.

### 4.2 Stem reserve mobilization

During the post-anthesis periods, the photosynthetic capacity of leaves declines due to natural senescence and various biotic and abiotic stresses. There is a rapid decline in the current leaf photosynthesis rate under terminal drought conditions (Johnson et al., 1981). Grain development in wheat depends upon carbohydrates from three sources: (i) carbohydrates produced before anthesis stored in the stem and remobilized to grains during grain filling, (ii) carbohydrates produced after anthesis and translocated directly to the grains, and (iii) carbohydrates produced after anthesis but stored temporarily in the stem before being remobilized to the grains (Gallagher et al., 1975; Daniels et al., 1982; Kobata et al., 1992). Thus, as the highest accumulation of water-soluble carbohydrates (WSCs) occurred at 12 DAA (Gurumurthy et al., 2023) and all the leaves including the flag leaves were defoliated at 12 DAA, spike weight difference between 12 DAA and at physiological maturity was most likely due to the mobilization of stored reserves from the stems to the grains.

The resulting higher expression of PPR5 and endoglucanse-8 like in the superior lines under drought stress in the peduncle at 12 DAA was probably to mobilize the stored carbon pool to the grain for sink requirements. This was further substantiated by the observation that the highest starch metabolism occurs at 14 days after anthesis in wheat (Cimini et al., 2015). The role of PPR proteins in inducing drought (Luo et al., 2022) and salt tolerance (Lu et al., 2022) in rice has been reported. The role of β-glucanase in plant development and adaptive response has also been stated (Perrot et al., 2022). In addition, the enhanced activity of ST protein kinase OSR1-like under drought stress in the superior lines was probably due to the fact that the ABA level enhanced the activity of the ST protein kinase for carbon mobilization. CaDIK1, an ST kinase, has been reported to regulate drought tolerance by modulating ABA sensitivity in pepper (Lim et al., 2020). Hu et al. (2022) reported that SnRK1 regulates the transport of non-structural carbohydrates from the sheath to the grain during grain filling in rice. Exposure to drought stress was shown to induce NCED gene expression in maize (Tan et al., 1997), tomato (Burbidge et al., 1999), Arabidopsis (Iuchi et al., 2001), and cowpea (Iuchi et al., 2000). The increase in the activity of NCED1 under drought stress might be due to the fact that the stress level enhanced the ABA level to provide stress tolerance to plants through fructans breakdown (Xiong and Zhu, 2003). Moreover, the role of ABA in SRM has also been demonstrated (Xu et al., 2016), indicating the role of ABA in the mobilization of temporarily stored carbon to grain under abiotic stress conditions, particularly under drought stress.

In this study, it was observed that drought stress enhanced the expression of key genes linked to SRM, thereby causing the mobilization of stored carbons in the stems to the grains.

### 4.3 1000-grain weight and spike weight difference

The combined effect of heat and drought stress significantly decreased the grain yield in all the RILs (Kumar et al., 2021; Meena et al., 2023). Drought and heat stress decrease the grain filling duration and grain filling rate, thereby reducing the grain yield (Prasad et al., 2008). However, drought stress enhanced the SWD due to a higher SRM (Gurumurthy et al., 2023; Ntawuguranayo et al., 2024). In the cluster analysis, it was shown that HDHI1113 and HDHI87 were grouped together with the highest cluster score. The PCA also revealed that most of the traits contributed towards the total variance, indicating a significant contribution of SG and SRM traits towards grain filling in wheat under stress conditions. The spider chart also clearly displays the significant contribution of SG and SRM traits to grain yield under stress conditions, which can be further exploited for yield improvements.

## 5 Conclusion

In this era of global climate change, there is an urgent need to develop new crop varieties to meet the ever-increasing demand for food. The identification of genomic regions regulating physiological traits will pave the way to breeding new and improved crop varieties. SG and SRM are crucial for grain filling in wheat during abiotic stress. In this study, we validated our mapped QTLs for SG and SRM by employing a gene expression and physiological approach, which can be used for MAS to improve the yield potential under stress conditions. By employing selection indexes, we selected two superior lines (HDHI113 and HDHI87) that can be used as suitable donor parents for SG and SRM traits in elite wheat cultivars. Moreover, the identified QTL-linked markers will help wheat breeders to accumulate desirable allelic combinations in future breeding programs. Thus, understanding the genetic basis of SG and SRM will improve crop productivity under abiotic stress conditions not only in wheat but in other cereal crops as well.

## Data Availability

The datasets presented in this study can be found in online repositories. The names of the repository/repositories and accession number(s) can be found in the article/**Supplementary Material**.
