# Supplementary material for: Validation of stay-green and stem reserve mobilization QTLs: physiological and gene expression approach
Source: Front Plant Sci. 2025 Feb 17;16:1541944. doi: 10.3389/fpls.2025.1541944 (PMC11873102; doi:10.3389/fpls.2025.1541944)
Supplement: Supplementary file 1 [file DataSheet1.zip › Tables.DOCX]

**Supplementary Table 1** Minimum and maximum temperatures during the cropping season of 2022-23 under control, drought, heat, and combined stress conditions

| **Treatment** | **Min. Temp. (°C)** | **Max. Temp. (°C)** |
| --- | --- | --- |
| Control | 8.0-16.4 | 23.0-34.0 |
| Drought | 8.0-16.4 | 23.0-34.0 |
| Heat | 13.6-17.8 | 24.6-34.5 |
| Combined | 13.6-17.8 | 24.6-34.5 |

**Supplementary Table 2** List of candidate genes and respective primers

| **Traits** | **Genes** | **Primers** |
| --- | --- | --- |
| **SPAD** | Chlorophyll a-b binding protein of LHCII type 1-like (TraesCS5B02G353200) | FP-GTTCTCCATGTTCGGCTTCTT  RP-CCCAGGCGTTGTTGTTGA |
|  | Photosystem II reaction center PSB28 protein (TraesCS5B02G516600) | FP-CGTCGATCCAGTTCATCCAG  RP-GCTGCTCGAAGGTGAAGAT |
|  | Chlorophyll synthase, chloroplastic (TraesCS1D02G226100) | FP-CTGGTCCATGTCTTACAGGATAC  RP-CCTGAAGGAATAGGACGATAAGG |
|  | 7-HCAR, chloroplastic-like (TraesCS1D02G241000) | FP-TCATCGCCTTCCTGCTAAATC  RP-CCGGTTCACATACAGGTAGTTC |
|  | Ribulose bisphosphate carboxylase (RUBISCO) small subunit (TraesCS2B02G079100) | FP-GCCGATTGAGGGTATCAAGAAG  RP-TTGGAGCGGATCAGGTAGT |
|  | L-ascorbate peroxidase 2, cytosolic (TraesCS2B02G096200) | FP-CAGGACATTGTTGCTCTTTCTG  RP-GTTGTCGAAGATCAAAGGATTGG |
|  | Pyrroline carboxylate synthase (TraesCS3B02G395900) | FP-CGAGAAGATGTACTGCCCATTAG  RP-TTTCCACTTCGGATTGCTAGAG |
|  | Cytokinin riboside 5'-monophosphate phosphoribohydrolase LOGL10 (TraesCS1A02G156100) | FP-CTCGAGAACTACGTGCCTTAC  RP-GTACCCTAGGTGCCCTATCT |
| **LSR** | Potassium transporter 9 (TraesCS2D02G106600) | FP-CAAGAAGTCTGGCTGGATGT  RP-CTGGATTGCACTGTAGGAGAAG |
|  | Glutamate decarboxylase 1-like (TraesCS4B02G052300) | FP-CGAGCTCATCTTCCACATCAA  RP-GCTGGTAATACTGCGCGATAA |
|  | Aspartyl protease family protein 2-like isoform X1 (TraesCS2D02G112800) | FP-GCTATGTTTCCTCCTTGCTTTG  RP-GTTGTGTGGTTGAGCTTGTG |
| **SRM** | PPR5 (TraesCS6B02G332800) | FP-CATACACCAGGGCTAACGATAA  RP- CCAGGGCATCAAGGAAGAA |
|  | Endoglucanase 8-like (TraesCS6B02G368000) | FP-CCCTGCACCTACATCAACTC  RP-TGATCGTATCCGTGGGACT |
|  | Serine/threonine-protein kinase OSR1-like (TraesCS6B02G386100) | FP-GGGAATGTCGAGTTGGATAAGG  RP-GCCTCAGCTGAAGATGGTATG |
|  | 9-cis-epoxycarotenoid dioxygenase NCED1, chloroplastic-like (TraesCS6B02G298800) | FP-ACAACAGGTTCGTGGTGATG  RP-TCGGCCTGAGTGACGAATA |
| **Reference gene** | Actin | FP- GTGCCCATTTACGAAGGATA  FP- GAAGACTCCATGCCGATCAT |

**Supplementary Table 3** **Photosynthetic pigments under control, drought, heat and combined stress condition**

| **Treatments** | Lines | Chlorophyll a  (mg/gDW) | Chlorophyll b  (mg/gDW) | Total Chlorophyll  (mg/gDW) | Total Carotenoid  (mg/gDW) |
| --- | --- | --- | --- | --- | --- |
| **Control** | HDHI113 | 8.33±0.062^a^ | 3.03±0.022^a^ | 11.36±0.04^a^ | 1.915±0.066^a^ |
|  | HDHI87 | 8.08±0.114^b^ | 2.90±0.075^a^ | 10.99±0.08^b^ | 1.913±0.028^a^ |
|  | HD3086 | 7.26±0.038^b^ | 2.70±0.019^b^ | 9.96±0.029^c^ | 1.81±0.047^ab^ |
|  | HI1500 | 6.82±0.028^c^ | 2.56±0.016^bc^ | 9.37±0.026^d^ | 1.67±0.065^b^ |
|  | HDHI185 | 6.51±0.186^c^ | 2.37±0.084^d^ | 8.88±0.126^e^ | 1.43±0.061^c^ |
|  | HDHI80 | 6.53±0.138^c^ | 2.40±0.054^cd^ | 8.93±0.084^e^ | 1.42±0.091^c^ |
| **Drought** | HDHI113 | 7.56±0.039^a^ | 2.96±0.069^a^ | 10.52±0.108^a^ | 1.80±0.024^a^ |
|  | HDHI87 | 7.34±0.053^b^ | 2.59±0.063^b^ | 9.93±0.056^b^ | 1.84±0.048^a^ |
|  | HD3086 | 6.87±0.019^c^ | 2.40±0.030^c^ | 9.26±0.014^c^ | 1.73±0.01^ab^ |
|  | HI1500 | 6.62±0.007^d^ | 2.17±0.003^d^ | 8.79±0.008^d^ | 1.61±0.062^b^ |
|  | HDHI185 | 6.03±0.035^e^ | 1.56±0.048^e^ | 7.59±0.037^e^ | 1.11±0.031^c^ |
|  | HDHI80 | 5.98±0.032^e^ | 1.64±0.019^e^ | 7.62±0.036^e^ | 1.11±0.050^c^ |
| **Heat** | HDHI113 | 7.29±0.082^a^ | 1.78±0.044^b^ | 9.07±0.063^a^ | 1.72±0.078^a^ |
|  | HDHI87 | 6.75±0.005^b^ | 1.99±0.039^a^ | 8.73±0.035^b^ | 1.68±0.004^a^ |
|  | HD3086 | 6.41±0.006^c^ | 1.78±0.007^b^ | 8.19±0.003^c^ | 1.64±0.003^a^ |
|  | HI1500 | 6.27±0.002^d^ | 1.67±0.017^b^ | 7.94±0.015^d^ | 1.63±0.006^a^ |
|  | HDHI185 | 5.39±0.059^e^ | 1.23±0.017^c^ | 6.62±0.068^e^ | 0.59±0.039^b^ |
|  | HDHI80 | 5.45±0.010^e^ | 1.26±0.087^c^ | 6.71±0.077^e^ | 0.57±0.024^b^ |
| **HD** | HDHI113 | 7.08±0.073^a^ | 2.78±0.129^a^ | 9.86±0.091^a^ | 1.36±0.104^a^ |
|  | HDHI87 | 6.62±0.029^b^ | 2.58±0.005^b^ | 9.19±0.035^b^ | 1.28±0.015^a^ |
|  | HD3086 | 5.98±0.116^c^ | 1.02±0.043^c^ | 6.99±0.073^c^ | 1.12±0.014^b^ |
|  | HI1500 | 5.25±0.017^d^ | 0.82±0.006^d^ | 6.06±0.011^d^ | 1.11±0.012^b^ |
|  | HDHI185 | 4.55±0.015^e^ | 0.49±0.009^e^ | 5.03±0.024^e^ | 0.41±0.005^c^ |
|  | HDHI80 | 4.58±0.039^e^ | 0.49±0.028^e^ | 5.07±0.066^e^ | 0.40±0.007^c^ |
| **LSD** | Control | 0.339 | 0.161 | 0.226 | 0.192 |
|  | Drought | 0.104 | 0.139 | 0.167 | 0.127 |
|  | Heat | 0.128 | 0.135 | 0.158 | 0.114 |
|  | HD | 0.184 | 0.174 | 0.177 | 0.134 |
|  | G×T | 0.194 | 0.141 | 0.170 | 0.134 |

*The different letters (as superscript) in a column denotes significant difference among the lines within the treatment according to LSD test at P* < *0.05. The value follows* ± *represent the standard error of mean (n* = *3).*

**Supplementary Table 4** **Photosynthetic traits, TSW and EWD under control, drought, heat and combined stress condition**

| Treatments | Lines | P_N_ (µmol CO_2_/m^2^/s) | g_sw_ (mol H_2_O/m^2^/s) | E (m mol H_2_O/m^2^/s) | WUE_i_ (µmol CO_2_/m mol H_2_O) | 1000 grain weight (g) | Spike weight difference (g) |
| --- | --- | --- | --- | --- | --- | --- | --- |
| **Control** | HDHI113 | 26.70±0.180^a^ | 0.48±0.002^a^ | 5.93±0.094^a^ | 4.51±0.091^a^ | 45.28±0.581^a^ | 0.99±0.051^a^ |
|  | HDHI87 | 25.78±0.202^a^ | 0.46±0.008^b^ | 5.83±0.079^a^ | 4.42±0.057**^a^** | 43.88±0.339^b^ | 0.91±0.015^ab^ |
|  | HD3086 | 24.09±0.235^b^ | 0.34±0.005^c^ | 5.52±0.112^b^ | 4.37±0.070^a^ | 41.49±0.217^c^ | 0.59±0.040^c^ |
|  | HI1500 | 23.84±0.042^b^ | 0.46±0.003^b^ | 5.45±0.018^bc^ | 4.38±0.017^a^ | 39.04±0.260^d^ | 0.86±0.016^b^ |
|  | HDHI185 | 20.28±0.565^c^ | 0.21±0.002^e^ | 5.30±0.081^c^ | 3.83±0.072^b^ | 36.66±0.291^e^ | 0.51±0.025^c^ |
|  | HDHI80 | 19.01±0.522^d^ | 0.32±0.002^d^ | 5.07±0.017^d^ | 3.75±0.099^b^ | 33.85±0.790^f^ | 0.39±0.013^d^ |
| **Drought** | HDHI113 | 24.06±0.300^a^ | 0.30±0.001^a^ | 5.20±0.066^a^ | 4.62±0.019^ab^ | 39.78±0.298^a^ | 1.73±0.037^a^ |
|  | HDHI87 | 23.01±0.546^ab^ | 0.25±0.002^b^ | 4.83±0.014^bc^ | 4.76±0.102^a^ | 39.72±0.144^a^ | 1.43±0.037^b^ |
|  | HD3086 | 21.94±0.494^b^ | 0.25±0.002^b^ | 4.96±0.013^b^ | 4.43±0.091^b^ | 39.08±0.294^a^ | 1.32±0.015^c^ |
|  | HI1500 | 21.58±0.410^b^ | 0.20±0.001^c^ | 4.92±0.035^b^ | 4.38±0.061^b^ | 36.48±0.605^b^ | 1.43±0.025^b^ |
|  | HDHI185 | 17.14±0.590^c^ | 0.15±0.001^e^ | 4.72±0.085^c^ | 3.63±0.121^c^ | 34.64±0.217^c^ | 1.15±0.014^d^ |
|  | HDHI80 | 15.95±0.669^c^ | 0.18±0.002^d^ | 4.73±0.039^c^ | 3.37±0.162^c^ | 31.51±0.177^d^ | 0.92±0.004^e^ |
| **Heat** | HDHI113 | 23.33±0.298^a^ | 0.26±0.003^b^ | 7.84±0.186^a^ | 2.98±0.064^a^ | 36.41±0.288^a^ | 0.63±0.025^a^ |
|  | HDHI87 | 19.84±0.397^b^ | 0.15±0.003^d^ | 6.55±0.164^cd^ | 3.04±0.058^a^ | 34.92±0.227^b^ | 0.61±0.021^a^ |
|  | HD3086 | 19.81±0.404^b^ | 0.24±0.002^c^ | 6.73±0.078^c^ | 2.94±0.072^a^ | 24.56±0.493^d^ | 0.42±0.020^c^ |
|  | HI1500 | 18.73±0.091^b^ | 0.41±0.015^a^ | 7.24±0.097^b^ | 2.59±0.038^b^ | 32.44±0.201^c^ | 0.51±0.016^b^ |
|  | HDHI185 | 14.38±0.645^d^ | 0.12±0.002^e^ | 5.38±0.224^e^ | 2.69±0.161^b^ | 21.45±0.396^e^ | 0.27±0.031^d^ |
|  | HDHI80 | 15.59±0.220^c^ | 0.23±0.005^c^ | 6.25±0.039^d^ | 2.49±0.029^b^ | 20.62±0.245^e^ | 0.23±0.022^d^ |
| **HD** | HDHI113 | 17.18±0.462^a^ | 0.25±0.003**^a^** | 5.36±0.067^a^ | 3.21±0.059^a^ | 31.13±0.804^a^ | 1.57±0.068^a^ |
|  | HDHI87 | 16.48±0.514^ab^ | 0.25±0.004^a^ | 5.39±0.127^a^ | 3.05±0.037^ab^ | 28.85±0.881^b^ | 1.34±0.019^b^ |
|  | HD3086 | 15.38±0.319^bc^ | 0.21±0.002^b^ | 4.98±0.110^b^ | 3.09±0.055^ab^ | 21.94±0.278^d^ | 1.24±0.014^c^ |
|  | HI1500 | 14.22±0.332^c^ | 0.19±0.003^c^ | 4.73±0.029^bc^ | 3.01±0.061^b^ | 23.81±0.451^c^ | 1.38±0.027^b^ |
|  | HDHI185 | 10.87±0.361^d^ | 0.16±0.002^d^ | 4.57±0.122^cd^ | 2.38±0.037^c^ | 18.89±0.427^e^ | 0.99±0.034^d^ |
|  | HDHI80 | 10.89±0.373^d^ | 0.17±0.002^d^ | 4.32±0.019^d^ | 2.52±0.077^c^ | 17.12±0.298^f^ | 0.88±0.020^e^ |
| **LSD** | **Control** | 1.012 | 0.011 | 0.222 | 0.212 | 1.346 | 0.088 |
|  | **Drought** | 1.505 | 0.005 | 0.144 | 0.300 | 0.952 | 0.073 |
|  | **Heat** | 1.119 | 0.020 | 0.427 | 0.241 | 0.949 | 0.067 |
|  | **HD** | 1.167 | 0.007 | 0.262 | 0.163 | 1.674 | 0.103 |
|  | **G×T** | 1.168 | 0.012 | 0.272 | 0.225 | 1.218 | 1.218 |

*The different letters (as superscript) in a column denotes significant difference among the lines within the treatment according to LSD test at P* < *0.05. The value follows* ± *represent the standard error of mean (n* =5).

Supplementary Table 5A Identification of clusters under control condition based on cluster scores

| Traits | *Relative importance | Cluster-I | Cluster-II | Cluster-III |
| --- | --- | --- | --- | --- |
| P_N_ | 1.000 | 26.231 | 23.957 | 19.643 |
| Gsw | 0.986 | 0.462 | 0.393 | 0.259 |
| E | 0.975 | 5.733 | 5.348 | 5.055 |
| WUE | 0.986 | 4.406 | 4.313 | 3.737 |
| TGW | 0.999 | 44.517 | 40.211 | 35.203 |
| SWD | 0.920 | 0.872 | 0.667 | 0.415 |
| Chl a | 1.000 | 8.204 | 7.038 | 6.520 |
| Chl b | 0.993 | 2.948 | 2.611 | 2.373 |
| Total Chl | 1.000 | 11.167 | 9.663 | 8.904 |
| Total Car | 0.965 | 1.848 | 1.681 | 1.376 |
| Total score |  | 106.389 | 95.882 | 83.484 |

Supplementary Table 5B Identification of clusters under drought stress condition based on cluster scores

| Traits | *Relative importance | Cluster-I | Cluster-II | Cluster-III |
| --- | --- | --- | --- | --- |
| P_N_ | 0.995 | 23.944 | 22.071 | 16.466 |
| Gsw | 0.998 | 0.297 | 0.229 | 0.169 |
| E | 0.985 | 5.128 | 4.833 | 4.661 |
| WUE | 0.995 | 4.601 | 4.501 | 3.484 |
| TGW | 0.948 | 37.714 | 36.429 | 31.353 |
| SWD | 0.983 | 1.702 | 1.371 | 1.015 |
| Chl a | 0.983 | 7.433 | 6.828 | 5.904 |
| Chl b | 0.998 | 2.957 | 2.380 | 1.600 |
| Total Chl | 0.993 | 10.442 | 9.258 | 7.549 |
| Total Car | 0.976 | 1.756 | 1.684 | 1.081 |
| Total score |  | 95.974 | 89.584 | 73.281 |

Supplementary Table 5C Identification of clusters under heat stress condition based on cluster scores

| Traits | *Relative importance | Cluster-I | Cluster-II | Cluster-III |
| --- | --- | --- | --- | --- |
| P_N_ | 0.984 | 20.646 | 18.426 | 14.742 |
| Gsw | 0.988 | 0.217 | 0.405 | 0.171 |
| E | 0.992 | 6.986 | 7.188 | 5.775 |
| WUE | 0.969 | 2.894 | 2.508 | 2.512 |
| TGW | 0.847 | 27.071 | 27.480 | 17.816 |
| SWD | 0.948 | 0.525 | 0.483 | 0.238 |
| Chl a | 1.000 | 6.815 | 6.272 | 5.422 |
| Chl b | 0.977 | 1.808 | 1.630 | 1.214 |
| Total Chl | 0.998 | 8.648 | 7.924 | 6.651 |
| Total Car | 0.981 | 1.648 | 1.596 | 0.567 |
| Total score |  | 77.258 | 73.912 | 55.108 |

Supplementary Table 5D Identification of clusters under combined stress condition based on cluster scores

| Traits | *Relative importance | Cluster-I | Cluster-II | Cluster-III |
| --- | --- | --- | --- | --- |
| P_N_ | 0.999 | 16.810 | 14.784 | 10.870 |
| Gsw | 0.998 | 0.251 | 0.202 | 0.164 |
| E | 0.968 | 5.199 | 4.696 | 4.302 |
| WUE | 0.987 | 3.091 | 3.010 | 2.417 |
| TGW | 0.997 | 29.893 | 22.802 | 17.949 |
| SWD | 0.996 | 1.454 | 1.305 | 0.932 |
| Chl a | 0.985 | 6.748 | 5.527 | 4.497 |
| Chl b | 0.993 | 2.660 | 0.910 | 0.484 |
| Total Chl | 0.995 | 9.480 | 6.495 | 5.028 |
| Total Car | 0.976 | 1.287 | 1.085 | 0.394 |
| Total score |  | 76.872 | 60.817 | 47.038 |
