# Supplementary material for: Validation of stay-green and stem reserve mobilization QTLs: physiological and gene expression approach
Source: Front Plant Sci. 2025 Feb 17;16:1541944. doi: 10.3389/fpls.2025.1541944 (PMC11873102; doi:10.3389/fpls.2025.1541944)
Supplement: Supplementary file 1 [file DataSheet1.zip › Figures.DOCX]

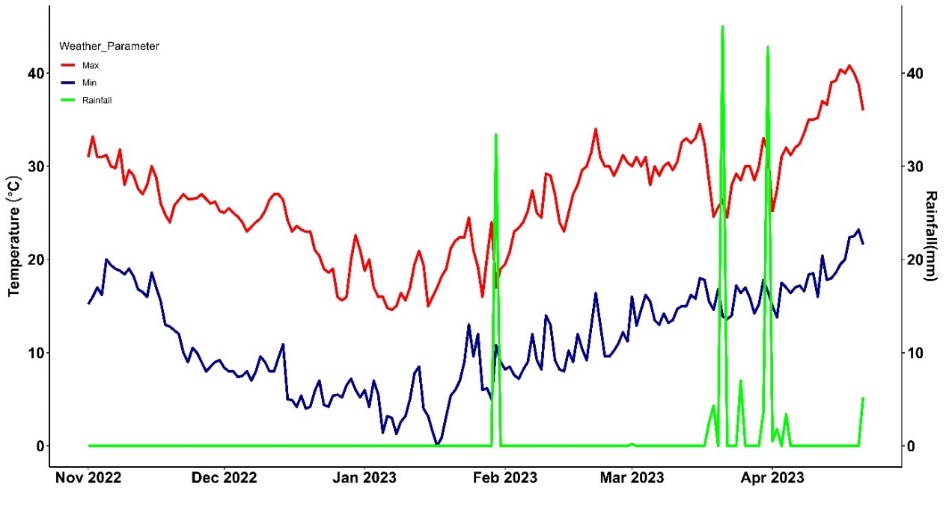


Supplementary Figure 1 Weather data for the cropping season of 2022-23

**
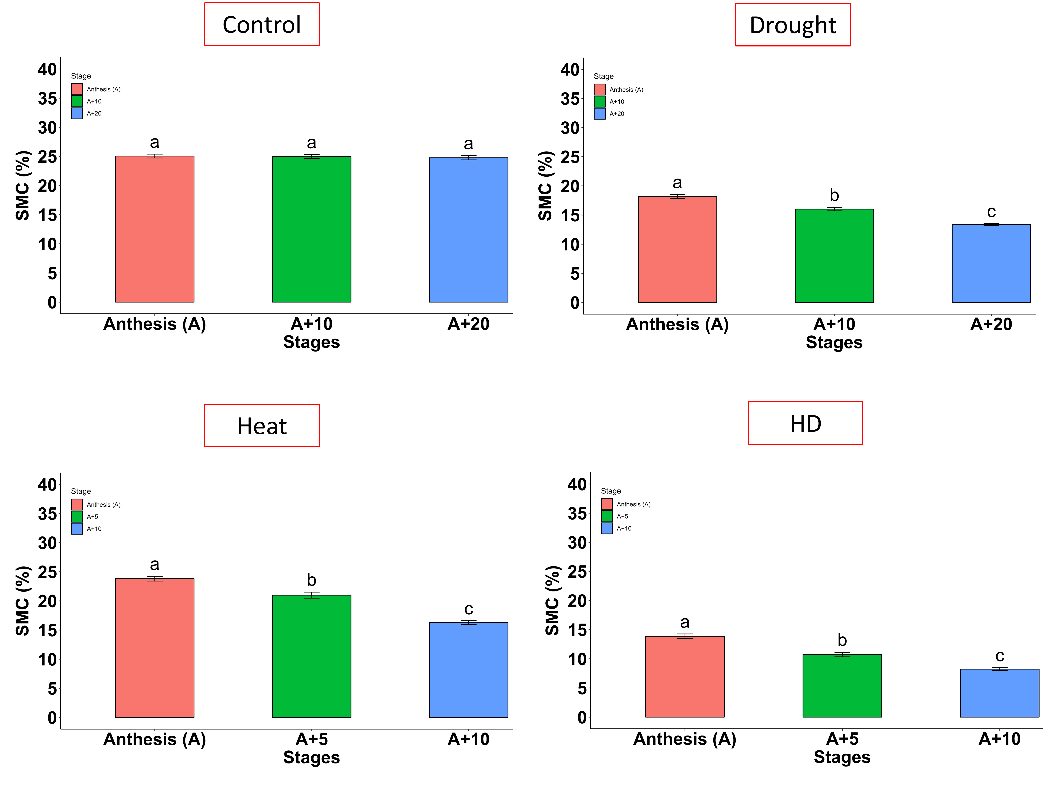
**

Supplementary Figure 2 Soil moisture (%) recorded during cropping season 2022-23


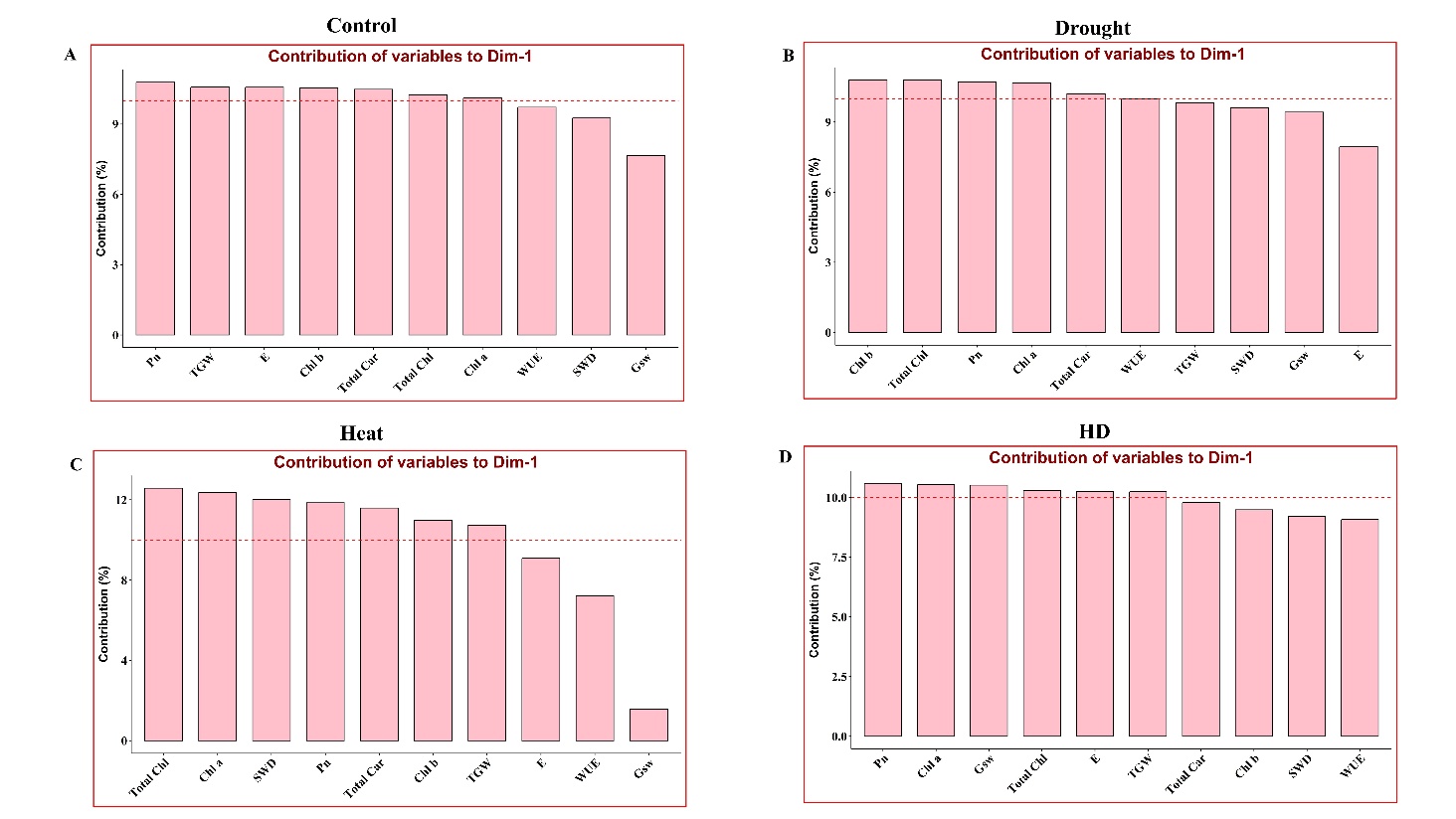


Supplementary Figure 3 Contribution of variables to Dimension-1 under control (A), drought (B), heat (C) and combined stress (D) condition.
